# Treatment for middle cerebral artery bifurcation aneurysms: in silico comparison of the novel Contour device and conventional flow-diverters

**DOI:** 10.1007/s10237-024-01829-3

**Published:** 2024-04-08

**Authors:** Mengzhe Lyu, Ryo Torii, Ce Liang, Thomas W. Peach, Pervinder Bhogal, Levansri Makalanda, Qiaoqiao Li, Yiannis Ventikos, Duanduan Chen

**Affiliations:** 1 Department of Mechanical Engineering, University College London, London, UK; 2 Department of Mechanical and Aerospace Engineering, Monash University, Clayton, Australia; 3 Department of Interventional Neuroradiology, The Royal London Hospital, London, UK; 4 School of Medical Technology, Beijing Institute of Technology, Beijing, 100081 China; 5 School of International Education, University of International Business and Economics, Beijing, 100029 China

**Keywords:** Middle cerebral artery bifurcation aneurysms, Contour device, Flow-diverters, Computational fluid dynamics (CFD)

## Abstract

Endovascular treatment has become the standard therapy for cerebral aneurysms, while the effective treatment for middle cerebral artery (MCA) bifurcation aneurysms remains a challenge. Current flow-diverting techniques with endovascular coils cover the aneurysm orifice as well as adjacent vessel branches, which may lead to branch occlusion. Novel endovascular flow disruptors, such as the Contour device (Cerus Endovascular), are of great potential to eliminate the risk of branch occlusion. However, there is a lack of valid comparison between novel flow disruptors and conventional (intraluminal) flow-diverters. In this study, two in silico MCA bifurcation aneurysm models were treated by specific Contour devices and flow-diverters using fast-deployment algorithms. Computational fluid dynamic simulations were used to examine the performance and efficiency of deployed devices. Hemodynamic parameters, including aneurysm inflow and wall shear stress, were compared among each Contour device, conventional flow-diverter, and untreated condition. Our results show that the placement of devices can effectively reduce the risk of aneurysm rupture, while the deployment of a Contour device causes more flow reduction than using flow-diverters (e.g. Silk Vista Baby). Besides, the Contour device presents the flow diversion capability of targeting the aneurysm neck without occluding the daughter vessel. In summary, the in silico aneurysm models presented in this study can serve as a powerful pre-planning tool for testing new treatment techniques, optimising device deployment, and predicting the performance in patient-specific aneurysm cases. Contour device is proved to be an effective treatment of MCA bifurcation aneurysms with less daughter vessel occlusion.

## Introduction

The effective treatment of middle cerebral artery (MCA) bifurcation aneurysms remains challenging, as several lesions present in such sites (Alfano et al. 2013; Zhang et al. 2018). Current treatment options focus either on aneurysm coil retention supported by a stent-like device positioned in the parent vascular lumen or on conventional (intraluminal) flow-diverters to disrupt blood flow within the aneurysm sac (Zhang et al. 2017; Mokin et al. 2020). One limitation of using conventional flow-diverters for such bifurcation aneurysms is that one daughter needs to be jailed, with evidence of causing branch occlusion and mandating antiplatelet medication (Raychev et al. 2016; Narata et al. 2018). Significant issues regarding jailed vessel occlusion in clinical cases have been reported in the literature. A study by (Saatchi et al. 2012) covered 46 ‘uncoilable’ aneurysms that originated at vessel bifurcations, with successful treatment by the *Pipeline*™ *Embolisation Device* (PED) reported in 41 (89.1%) cases. Among the remaining 5 cases (10.9% of the total), 1 patient died due to complete occlusion of the jailed daughter vessel. Similar results were reported by (Saleme et al. 2014). A retrospective analysis of 37 cases of bifurcation aneurysms treated with flow-diverters. At 6-month follow-up, complete aneurysm occlusion was observed in 97.3% of cases, while the new permanent neurological deficit was observed in 9.4% of patients. During follow-up, jailed daughter vessel occlusion was found in 32.4% of aneurysms and 40.5% of cases showed significant vessel stenosis. However, the effects of vessel occlusion or narrowing were only symptomatic in 13.5% of all aneurysms.

**Fig. 1:**
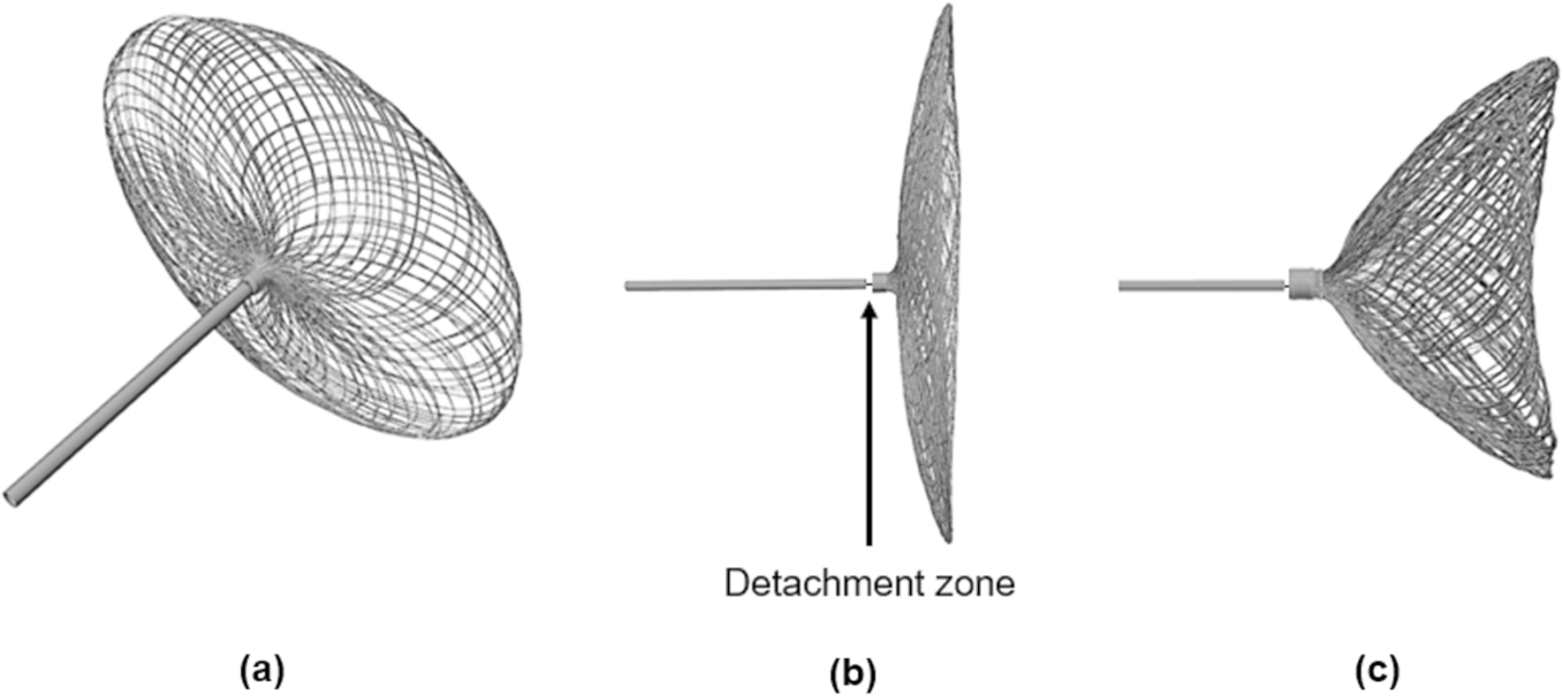
Overview of the Contour device, a dual-layer circular braided structure (**a**) with its proximal platinum marker connected to the introducer wire at the detachment zone (**b**). Inside the aneurysm (**c**), it typically adapts to the lower half of the sphere with its largest diameter at the equator level

As an alternative, intravascular devices create a barrier at the neck inside the aneurysm, which potentially eliminates the risk of branch occlusion and the need for long-term anticoagulation therapy (Pierot et al. 2012; Liebig et al. 2015; Arthur et al. 2019; Youssef et al. 2021). Endovascular flow disruptors include the Woven-EndoBridge (WEB, MicroVention) (Goyal et al. 2020), the LUNA-AED (Medtronic) (Piotin et al. 2018), and, most recently reported, the Contour (Cerus Endovascular) (Akhunbay-Fudge et al. 2020; Bhogal et al. 2021). Both the WEB and LUNA-AED devices feature a spherical braid that fills the aneurysm dome, while the Contour is built on a planar braid (Bhogal et al. 2019), entirely positioned at the neck (see Fig. 1).

The Contour Neurovascular System™ (Cerus Endovascular Inc., Fremont, CA) is designed to target the neck of aneurysms by providing flow disruption within the aneurysm sac combined flow diversion at the neck of the aneurysm (Thormann et al. 2022). This Contour is a circular, dual-layered structure of 2 × 72 nitinol wires (Fig. 1a) with 1 platinum marker (Fig. 1b). It is radiopaque and retrievable until electrolytically detached. On deployment, the device adapts to the lower half of the aneurysm, covering the neck (Fig. 1c). The Contour is deployed through a 0.027 microcatheter (″). Sizing is performed relative to the aneurysm neck width and diameter, disregarding height, with 5-, 7-, 9-, and 11-mm-diameter devices available (Liebig et al. 2022). Moreover, the device is designed to support neo-intimal growth across the mesh at the neck after thrombosis of the aneurysm.

Recent data from the CERUS study (the first multicentre clinical trial evaluating safety and effectiveness outcomes with the Contour neurovascular system) have indicated promising results in the treatment of unruptured intracranial bifurcation aneurysms (Lawson et al. 2017; Biondi et al. 2023). To further evaluate the performance of Contour and its differences compared to conventional flow-diverters when treating bifurcation aneurysms, patient-specific computational fluid dynamics (CFD) models can be utilised to evaluate the effects of novel Contour device.

In this work, the Contour devices were deployed in two MCA bifurcation aneurysm in silico models and then compared with conventional endovascular flow-diverter devices deployed in the same model. The structure of the Contour device was acquired via micro-computed tomography (CT) scanning. This study evaluates two important hemodynamic parameters in the context of aneurysms, such as wall shear stress (WSS) and aneurysm inflow rate (*Q*).

## Methods

### In silico aneurysm models and clinical approach

**Fig. 2:**
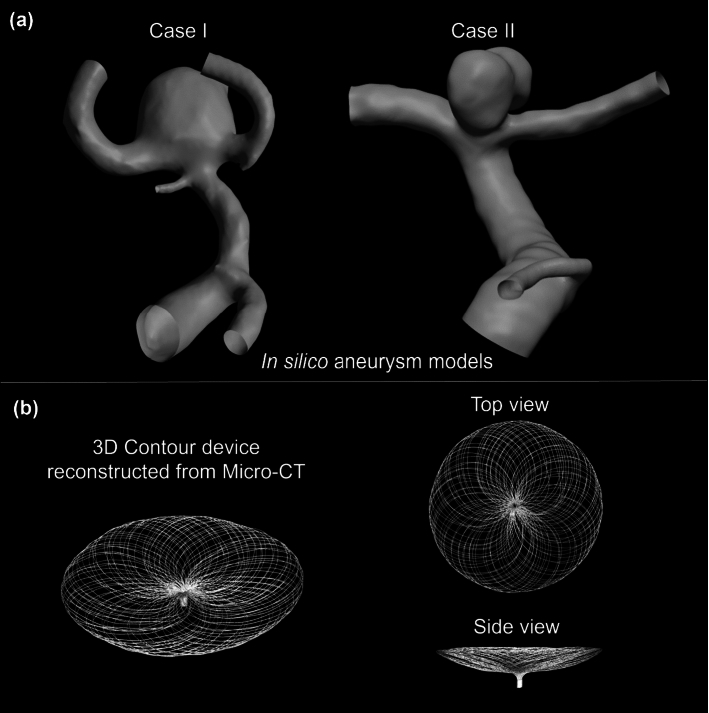
**a** In silico aneurysm models (Cases I and II) reconstructed from CT angiography at the pre-intervention stage. **b** Overview of the Contour device reconstructed from Micro-CT scan

Two in silico MCA bifurcation aneurysms models were manually segmented in OsiriX (OsiriX v.4.1.1, Freeware) from CT angiography imaging data. Both pre-intervention models were further reconstructed and trimmed (Fig. 2a). The structure of the Contour device was obtained from a Micro-CT scan (Fig. 2b). A conventional flow-diverter device was reconstructed in a range of sizes as per the methodology previously detailed in our previous study (Lyu et al. 2021). In the clinical approach, the selection of the sizing process of the Contour device is based on analysing aneurysm neck width (*d*_n_) and maximum aneurysm width (*d*_a_).

**Fig. 3:**
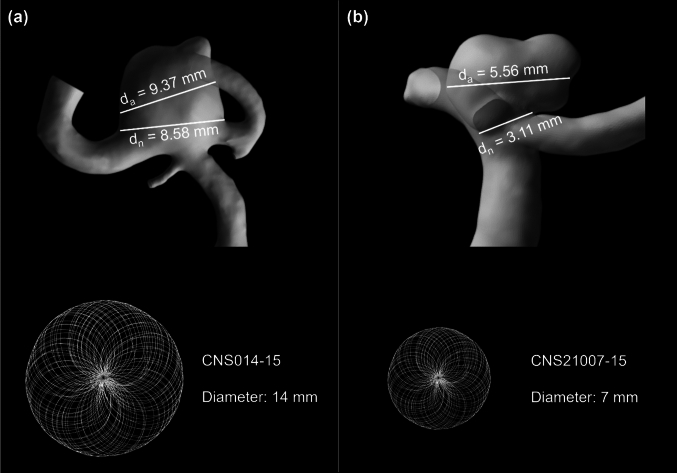
Selection of suitable Contour devices for Case I **a** and Case II **b** according to the neck width (*d*_n_) and maximum aneurysm width (*d*_a_) of the in silico aneurysm model

As can be seen in Fig. 3, the aneurysm neck and aneurysm widths are 8.58 mm and 9.37 mm, respectively, in Case I; therefore, a CNS014-15 (diameter: 14 mm) Contour device and Silk Vista Baby (SVB) (2.25 × 15 mm) were chosen for the deployment. Both sizes are in line with manufacturer instructions for use. As for Case II, the aneurysm neck and aneurysm width are 3.11 mm and 5.56 mm, respectively; therefore, a CNS21007-15 (diameter: 7 mm) Contour device and 3 × 15 mm SVB were chosen for the deployment inside the sac of the aneurysm.

### Virtual deployment

**Fig. 4:**
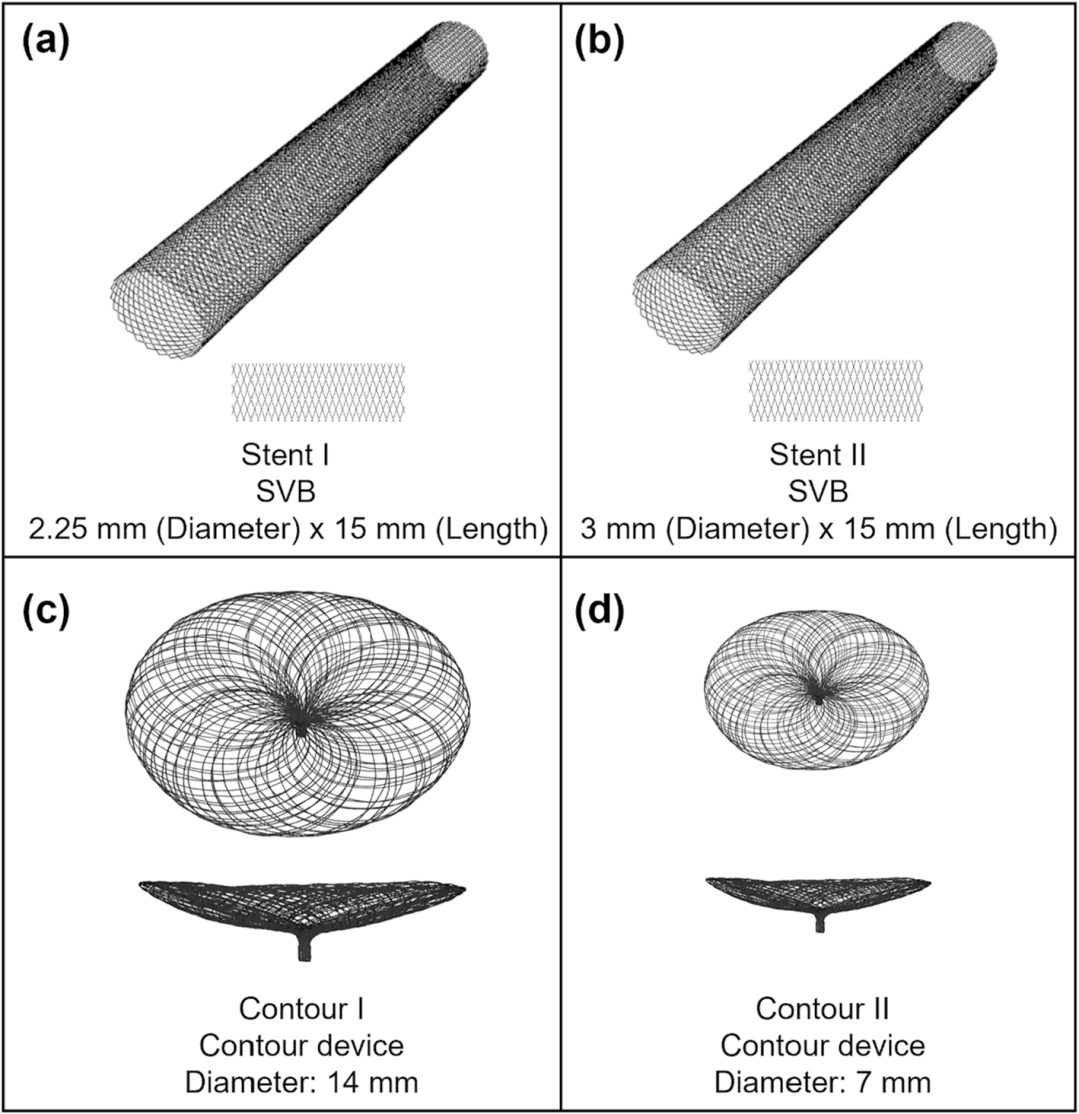
Devices designs to be deployed in each in silico aneurysm model, including Conventional flow-diverting stents (SVB) for Case I (**a**) and Case II (**b**), and Contour devices for Case I (**c**) and Case II (**d**). Note that the key dimensions of the above devices are listed at the bottom of each figure

To virtually model the deployment of both the Contour and SVB devices, four variants of the devices were created (Fig. 4). Stents I and II mimic the SVB devices (2.25 × 15 mm) and (3 × 15 mm) deployed as per manufacturer instructions with a free expansion of up to 2.25 mm and 3 mm in diameter. Contours I and II mimic the Contour device deployed in the neck of the aneurysm, and the size of the Contour device is based on the relative size of the aneurysm and specifications of the Contour device (Table 1).

**Table 1:** Contour device product selection table, CNS014-15 is selected for Case I, whereas CNS21007-15 is selected for Case II

Product code	Inner diameter of microcatheter (in)	Diameter (mm)	Aneurysm neck width, *d*_n_ (mm)	Maximum aneurysm width, *d*_a_ (mm)
CNS21005-15	0.021”	5	2.0–3.0	2.0–3.5
CNS21007-15	0.021”	7	3.0–5.0	3.0–5.5
CNS21009-15	0.021”	9	4.0–6.0	5.0–7.5
CNS011-15	0.027”	11	5.0–8.0	7.0–8.5
CNS014-15	0.027”	14	7.0–10.0	8.0–10.5

*1 in equal to 25.4 mm

A fast virtual deployment algorithm was used for device placement. Deployment algorithms were reported in our previous study (Peach et al. 2014a; Spranger and Ventikos 2014; Lyu et al. 2021). For SVB, a representative device wireframe is compressed to minimise the diameters and then aligned with the vessel centreline to simulate the device sheathing process. The device unsheathing process is accomplished by the SVB releasing progressively along its length. Within the limit of the vessel wall, the device extends to a stress-free shape. As for the Contour device, the wireframe is obtained from a Micro-CT scan and then compressed to reduce the diameter and align with a deployment path into an aneurysm to mimic the sheathing process. The device unsheathing is again achieved by relaxing the device, which then expands to its original shape inside the aneurysm. By relaxing the device, the unsheathing process is achieved by the device expanding to its original shape inside the aneurysm. Device sizing, porosity, and pore density are shown in Table 2 for the SVB and Contour device as per manufacturers’ guidance (Eker et al. 2022).

**Table 2:** Contour device product selection table, CNS014-15 is selected for Case I, whereas CNS21007-15 is selected for Case II

	Device	Typical porosity (deployed) (%)	Typical pore density (deployed) (mm^−1^)
Stent I	SVB	60	45
Stent II	SVB	60	45
Contour I	Contour	/	/
Contour II	Contour	/	/

### CFD simulation

Blood was modelled as an incompressible fluid, and the 3D Navier–Stokes equations were solved in CFD-ACE + (ESI Group, Paris, France) using the finite volume method with a central differencing scheme for spatial interpolations. We have used a second-order geometry-weighted interpolation for the convection terms of the momentum conservation equation, and we used pure 2nd order central finite differences both for the diffusion terms and for all relevant derivatives in the pressure correction equation. The SIMPLE Consistent (SIMPLEC) pressure correction method (Van Doormaal and Raithby 1984; Lonsdale 1993) and an algebraic multigrid method for convergence acceleration were used (Ni and Abdou 2007). Although previous studies (Valencia and Solis 2006; Liu et al. 2021) have confirmed that the non-Newtonian effect of blood in the brain is small in the cerebral circulation, blood is modelled as a Newtonian fluid with a density of 1000 kg/m^3^ and a dynamic viscosity of 0.004 Pa·s as many well-known CFD studies of cerebrovascular blood flow studies (Xu et al. 2018; Hossain et al. 2021; Boite et al. 2023). Rigid wall assumption was applied to the arterial walls (Xu et al. 2018; Hossain et al. 2021; Boite et al. 2023), with the effect of such an assumption on flow patterns having been shown to be small for the cerebral vasculature. Both the device struts and vessel walls were applied as no-slip boundary conditions. Convergence criteria of absolute or relative residual reduction to 1 × 10^−8^ and 1 × 10^−5^ were employed for all variables. The solution was considered to be converged if the change of residual is smaller than 1 × 10^−5^ (relative residual reduction) or the residual is below 1 × 10^−8^ (absolute residual). The convergence is typically reached in fewer than 100 iterations.

A radially symmetric inlet velocity boundary condition was applied to each geometry scaling the corresponding velocity to a mean internal carotid artery (ICA) flow rate of 230 ml/min (Valencia et al. 2006). A fixed pressure outlet boundary condition was applied to all geometry outlets; more complex outflow conditions incorporating windkessel models were considered but rejected given very little variation in daughter vessel flowrates under a constant pressure condition when compared to physiological values (to within 5% of mean flow rates reported in the literature). One of the objectives of this study is to find a balance between time and accuracy, therefore steady-state simulations were run on 32 × 2.80 GHz CPUs 128 GB RAM for 1–1.5 h depending on mesh size. Our previous study (Peach et al. 2017, 2019) reported that around 75 h is required for a transient simulation.

### Mesh Independence

**Fig. 5:**
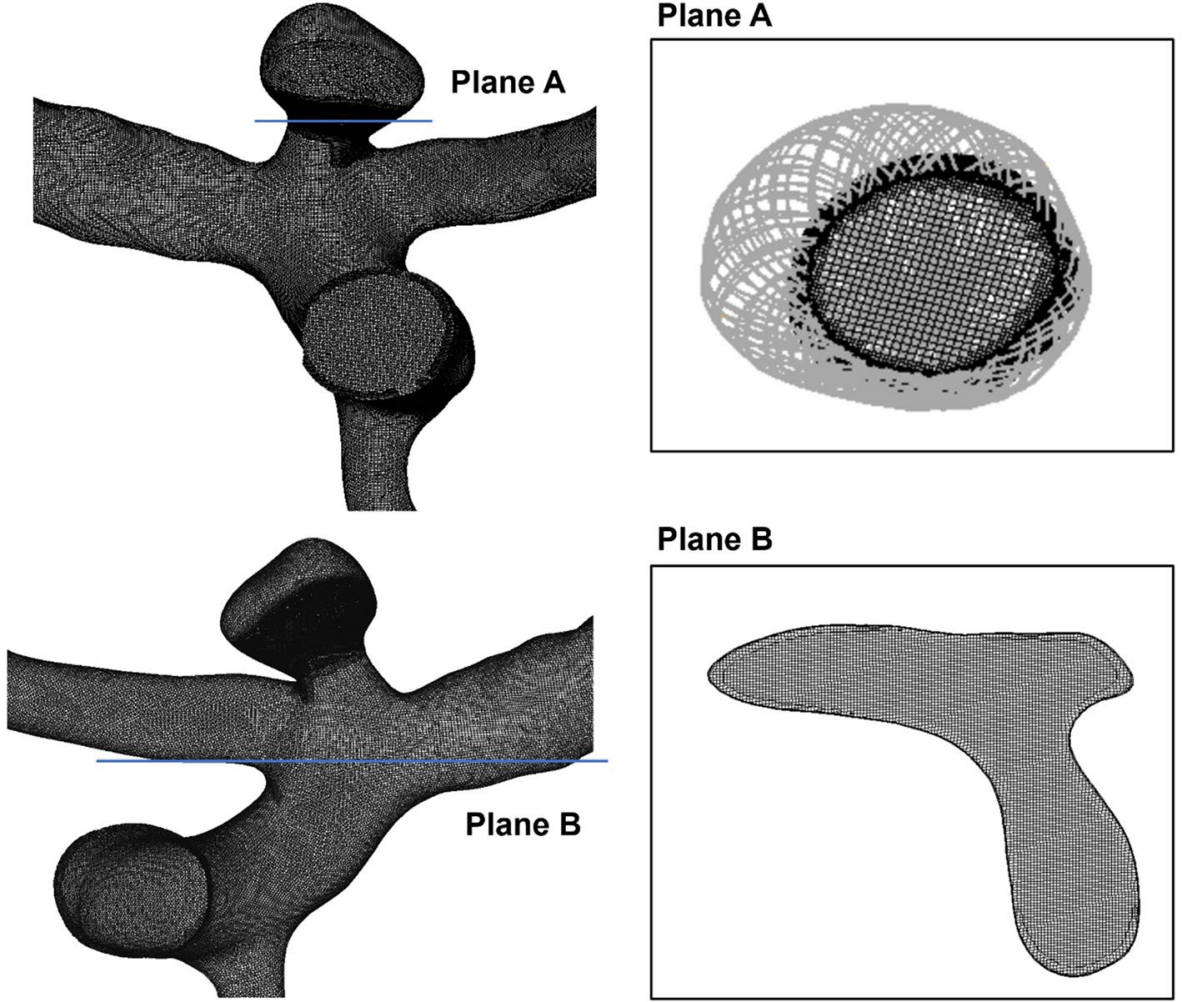
Details of mesh around the structure of the Contour device, a plane (Plane A) is used to cut interest region around the device and display the mesh around the Contour device structure. Detail of mesh at the vessel fluid domain is shown in Plane B

The middle cerebral artery (MCA) bifurcation aneurysm models, both without and with deployed devices, were meshed in CFD-VisCART (ESI Group, Paris, France). The meshing of each geometry is completed with a Projected Single Domain con-conforming mesh, an Omnitree Cartesian tree type, and three near-wall Cartesian layers to give a smooth and well-resolved boundary definition (Peach et al. 2016). The detail of mesh around the Contour device and the fluid domain of Case II is shown in Fig. 5.

As reported previously by our group (Peach et al. 2016), simulations run for mesh independence achieved with a typical minimum cell density finer than 4000 elements/mm^3^ for Cases I and II; such mesh densities meet the requirements discussed in the literature for smooth velocity and WSS resolution (Stuhne and Steinman 2004; Valen-Sendstad and Steinman 2014) and are more than the mesh-independent values calculated for cerebral aneurysms both with and without devices deployed previously obtained by the authors (Peach et al. 2014a, b). Additionally, at this level, the number of mesh elements in each geometry measurement plane exceeds the recommendation of Jou et al*.* that is required to fully resolve flow features (Jou and Mawad 2011). The minimum cell densities of meshes are generated from 1 to 50,000 elements/mm^3^ to increase the levels of fineness. Aneurysm inflow is measured through a plane defined at the aneurysm neck. Mesh independence to within 1% for both aneurysm inflow (*Q*) and wall shear stress (WSS) magnitude was achieved, by meshing the geometries with a mesh density > 4000 elements per mm^3^.

CFD simulation results were post-processed in CFD-VIEW (ESI Group, Paris, France), by visualising the velocity streamlines and WSS distributions inside the aneurysm dome. Two planes are used to evaluate to monitor inflow: One is a neck plane placed proximal to the devices deployed that define the aneurysm boundary, and another plane is placed on the proximal daughter vessel perpendicular to the daughter vessel wall (Peach et al. 2017, 2019). WSS max is defined as the largest value of WSS in the region of interest, referring to the aneurysm sac or the aneurysm neck.

## Results and discussion

### Virtual deployment of devices

**Fig. 6:**
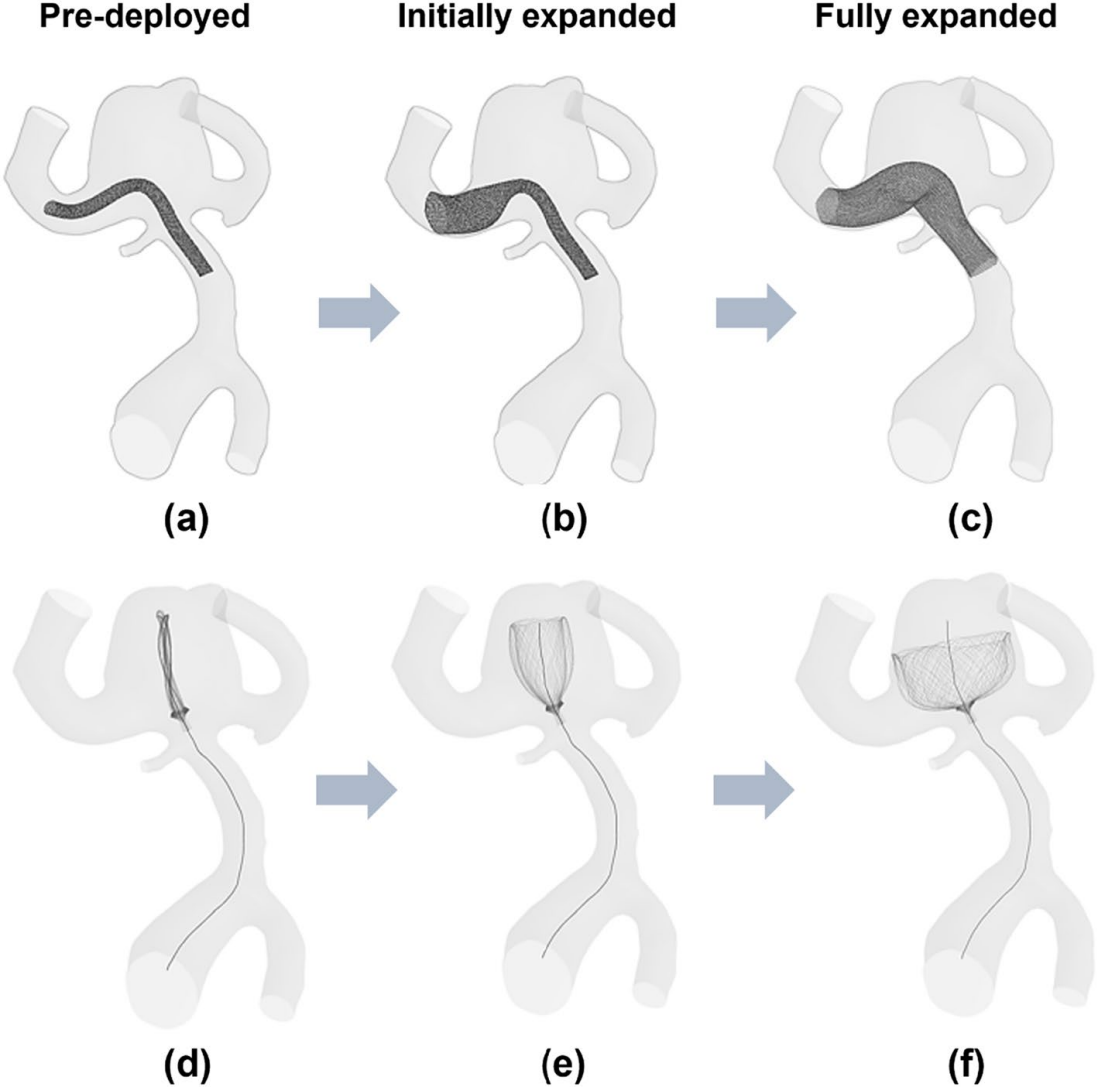
Virtual stenting procedure in Case I. **a** Crimped SVB flow-diverter placed on the centreline of the target vessel. **b** The initial stage of the SVB expansion process starts from the distal to the proximal side of the device with respect to the aneurysm sac. **c** Configuration of fully expanded SVB. **d** Crimped Contour device aligned on the deployment path. **e** The initial stage of the Contour device expansion process. **f** Configuration of fully expanded Contour device

**Fig. 7:**
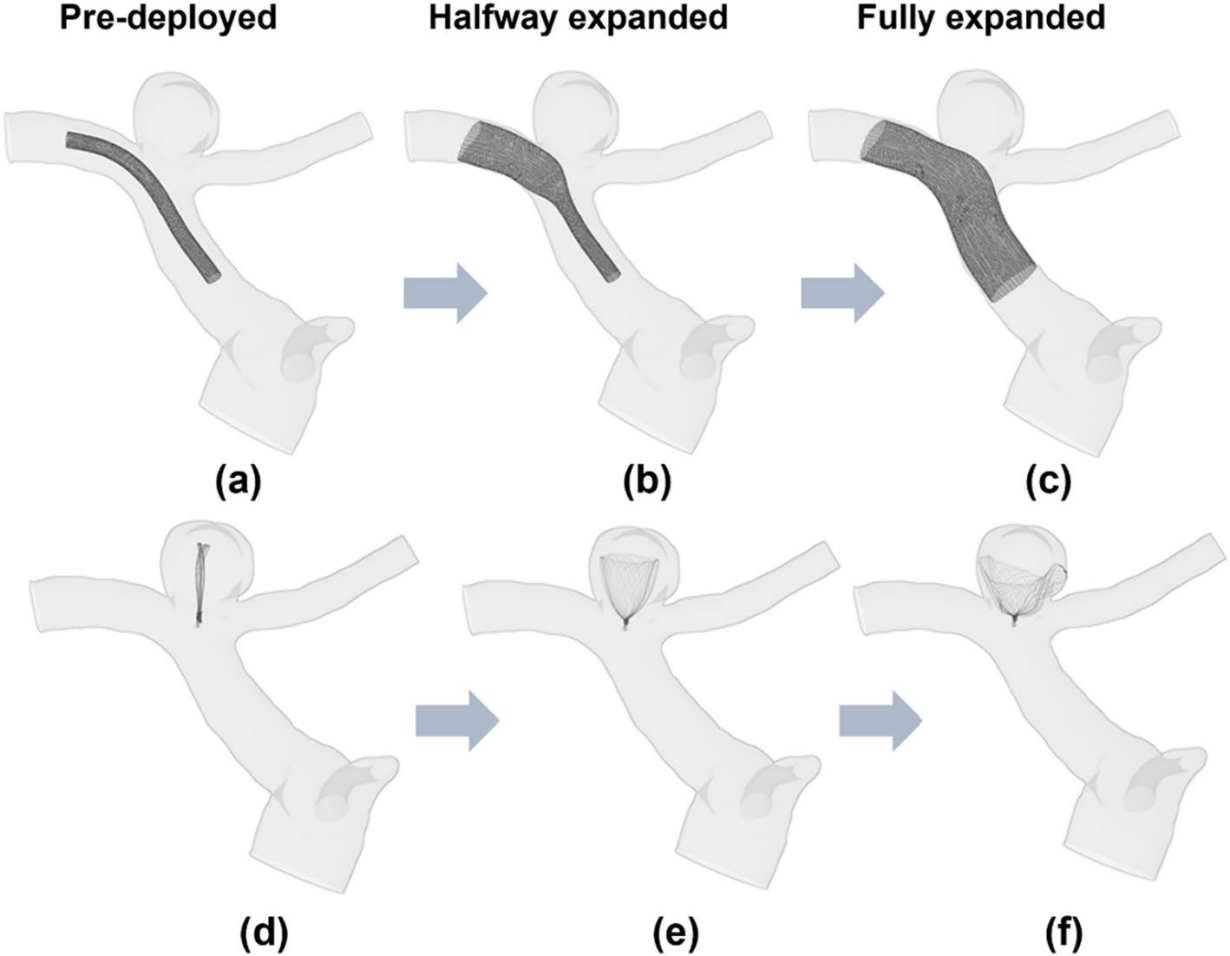
Virtual stenting procedure in Case II. **a** A crimped SVB flow-diverter was placed on the centreline of the target vessel. **b** Halfway through the SVB expansion process, starting from the distal to the proximal side of the device with respect to the aneurysm sac. **c** Configuration of fully expanded SVB. **d** Crimped Contour device aligned on the deployment path. **e** Halfway through the Contour device expansion process. **f** Configuration of fully expanded Contour device

The virtual fast stenting algorithm is applied to all devices (Stents I–II and Contours I–II) in two patient-specific aneurysm models, as shown in Table 2, Figs. 6 and 7. Similar to the clinical procedure, placement of the flow-diverter begins from distal side to proximal of the device with respect to the aneurysm sac. Figures 6 and 7 show the overall configurations of the SVB and the Contour device for Case I and Case II at the three stages of the deployment process.

The fast virtual stenting algorithms are developed in Visual Studio 2019 (Microsoft, Albuquerque, New Mexico, USA) running on a single 2.60 GHz core without any parallelisation (i.e. multithreading). After approximately 48 iterations, each device in each model reached the deployed position, with a computation time of less than 1 min per case. The device deployed made good contact with the vessel wall, and the vessel wall was considered fixed during the deployment process.

### Post- and pre-intervention hemodynamics

**Fig. 8:**
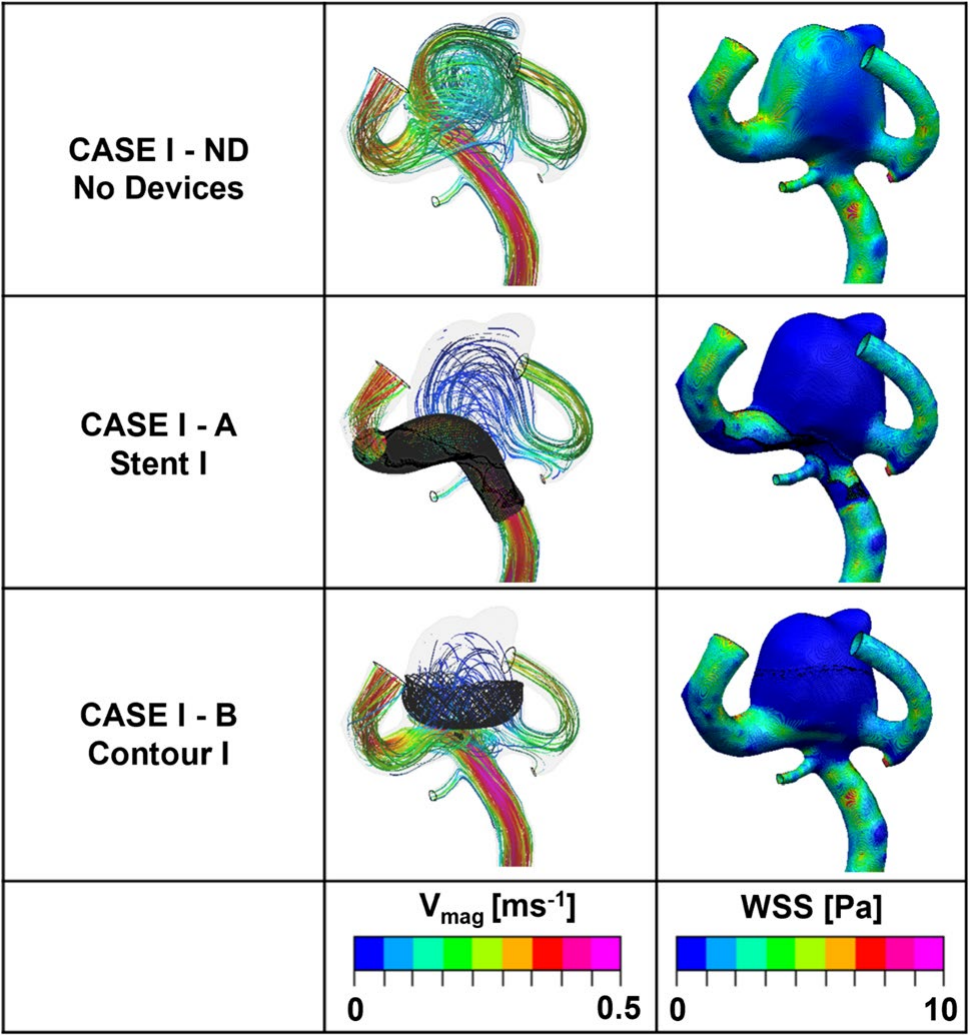
Velocity streamlines and WSS distributions for the No Device (ND) and selected devices (Stent I or Contour I) in the Case I model

**Fig. 9:**
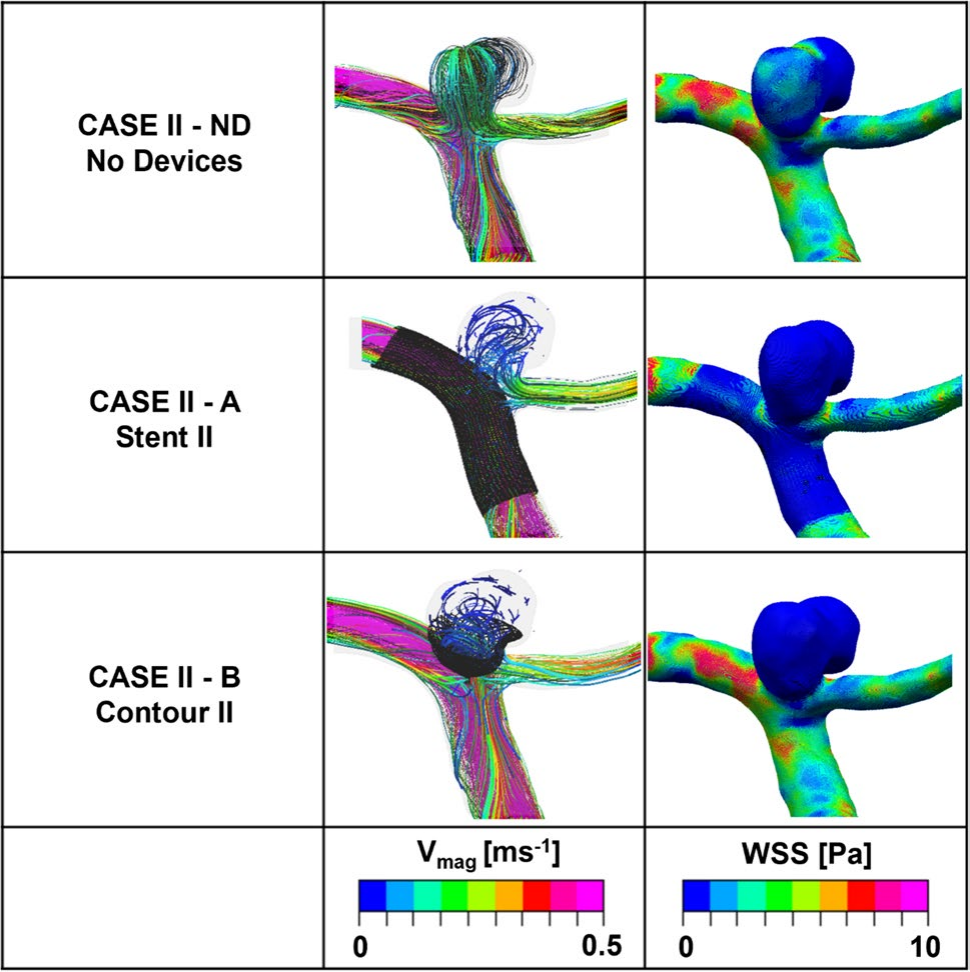
Velocity streamlines and WSS distributions for the No Device (ND) and selected devices (Stent II or Contour II) for the Case II model

Calculations of inflow entering (*Q*_in_) through the aneurysm neck with no device deployed in each case are shown in Table 3 with values of 126.4 and 51.8 ml/min, respectively, representing approximately 100% and 69.63%, respectively, of the parent vessel (MCA) average flowrate. Velocity streamlines for No Device cases are shown in the top rows of Figs. 8 and 9.

**Table 3:** Percentage reductions in total flow entering (*Q*_in_) the sac of Case I and Case II with different deployment devices

ID	Description	*Q*_in_ (ml/min)	% Reduction
Case I			
ND	No devices	126.4	–
A	Stent I	16.5	86.9
B	Contour I	11.2	91.1
Case II			
ND	No devices	51.8	–
A	Stent II	5.7	89.0
B	Contour II	4.5	91.4

In Case I, a relatively fast flow ($\sim$ 0.5 ms^−1^) entered deeply into the aneurysm dome, whereas the flow at a slower speed ($\sim$ 0.24 ms^−1^) was found at the entrance of the aneurysm dome in Case II. Most prominent in Case I, the jets of flow cause a region of increased WSS magnitude inside the dome of the aneurysm by the impact of the jet at the vessel wall of the aneurysm tip and the impingement flow leaving the neck of the aneurysm dome. The risk of aneurysm rupture is correlated with concentrated jet inflow, high aneurysm inflow rates, and regions of flow impingement and confirms the fragile nature of these particular aneurysm cases prior to intervention (Hassan et al. 2005; Castro et al. 2009; Doddasomayajula et al. 2017).

In Case I, a significant reduction in inflow entering the aneurysm sac by around 89.8% after the deployment of both devices (Table 3). The inflow reduction has been accomplished by eliminating the inflow jet almost completely and by decreasing the flow velocity that does enter the aneurysm substantially (second row, Fig. 8).

Compared to the performance of the SVB, the Contour device shows a slightly better inflow reduction ability with a reduction of 91.1% inflow entering the dome of the aneurysm (Table 3). Also, this device almost eliminates the aneurysm inflow jet with only isolated flow streamlines entering the left-hand side of the aneurysm (third row, Fig. 8). Besides, the velocity streamlines of Case I (A and B) further demonstrate that the right-hand daughter vessel serves as the primary outlet for the majority of the flow entering the aneurysm dome for both devices.

The change of flow pattern has effectively resolved a significant portion of the flow impingement observed in the No Device case, where a portion of the flow exits aneurysm through the left-hand daughter vessel resulting in elevated WSS max ($\sim$ 8 Pa) at the neck of the aneurysm. The WSS magnitude plot for Case I-A in Fig. 8 confirms that the introduction of the device has eliminated the flow jet and impingement region entirely. Consequently, the WSS throughout the entire aneurysm dome remains at approximately 2 Pa, which is a typical value for healthy blood vessels.

There are similarities in the performance of both devices for Case II as summarised in Table 3. Deployment of the SVB and Contour devices again results in an inflow reduction of around 90%. When comparing the first and second rows of Fig. 9, it can be observed that the results obtained from the SVB are similar to the previous findings. The presence of jets (> 0.25 ms^−1^) entering the aneurysm is reduced. The complex and impinging flow within the aneurysm dome is now transformed into a relatively simple circulating flow pattern, where blood enters the aneurysm from the right-hand side and exits through the left-hand side. Notably, the high WSS (max magnitude > 8 Pa) concentrated region seen close to the amorphous neck of the aneurysm in the No Device case is reduced dramatically after the device placements of SVB and the Contour device. However, the WSS max magnitude remains high in the left-hand daughter vessel after placement of the Contour device, whereas the WSS max magnitude is reduced to an average of 2 Pa in the region of the SVB placement location in the left-hand daughter vessel. Similar to Case I, the elimination of increased WSS region and impinging flow would suggest that both devices substantially reduce the rupture risk of aneurysm and facilitate aneurysm stabilisation and thrombosis.

### The effect on the daughter vessel

Table 4 shows a 20.60% increase in the daughter vessel flow rate after Contour device deployment, whereas there is a 15.38% decrease in the daughter vessel flow rate when SVB is deployed. As for Case II, there is a 3.37% increase in the daughter vessel flow rate after Contour deployment compared to an appropriately 1.81% decrease in the daughter vessel flow rate. The average daughter vessel flow rate increased by 11.99% for the Contour device and the average flow reduction in daughter vessel flow rate is 8.60% for the SVB device.

**Table 4:** Percentage reductions in total flow entering (*Q*_in-DV_) to the daughter vessel (DV) of Case I and Case II with different deployment devices

	Case I	Case II
ND (*Q*_in-DV,_ ml/min)	23.01	36.45
Contour (*Q*_in-DV,_ ml/min)	27.75	37.68
Relative change (%)	+ 20.60%	+ 3.37%
SVB (*Q*_in-DV_ ml/min)	19.47	35.79
Relative change (%)	−15.38%	−1.81%

The relative change (%) refers to the change in *Q*_in-DV_ of deploying Contour device over No devices (ND), and Silk Vista Baby (SVB) over No devices (ND)

An average change of 2.59% is observed in the non-stented daughter vessel flow rate after the deployment of both the Contour device and SVB in Case II, whereas there is a significant change (average change of 17.99%) in the non-stented daughter vessel flow rate after the deployment of both devices in Case I. This indicates that the aneurysm morphology might play a vital role in affecting fluid alternation to the device implantation. The large non-stented daughter vessel diameter ratios (SDRs; ratios between non-stented daughter vessel and main vessel diameters), small bifurcating angles (angle between main and non-stented daughter vessel) in Case I might contribute to the significant change in the non-stented daughter vessel flow rate.

Additionally, the average Relative Change (%) after the deployment of the Contour device is around 11.99%, whereas the average Relative Change (%) is reduced by 8.60% after the implantation of SVB. One possible cause is that the Contour device tends to “cover” the neck of an aneurysm which leads to more flow into the non-stented daughter vessel. On the other hand, SVB tends to lead more flow into the stented daughter vessel. This result illustrates that the Contour device offers the possibility of a flow diversion effect at the aneurysm neck, without the drawbacks of daughter vessel occlusion. However, without incorporating biochemical models to capture device endothelialisation the actual occlusion of the daughter vessel cannot be predicted.

There are several limitations to this study. First, the number of cases used in this proof-of-concept study is modest, with the future improvement in the automated 3D reconstruction algorithm and simulation pipeline more cases are warranted. Second, the movement of vessels during the cardiac cycle was not considered. (4D simulation) with time makes the simulation realistic at the expense of computational time. Third, the use of average flow rates and fixed pressure outlet boundary condition may be considered unrealistic, and certainly it reduces the patient-specific nature of the simulations. The Windkessel model can be introduced to facilitate the "correction" of left-to-right vessel flow imbalance which can improve the accuracy of the flow model. A time-dependent solution can provide more hemodynamic parameters such as Relative Residence Time (RRT) for the analysis of successful aneurysm occlusion, but as a trade-off, the computation time will increase significantly. Extending this study to include proper pulsatile flow simulations will certainly improve the accuracy and relevance of the implantation effect predictions, and it is a straightforward step, as it has been demonstrated in the past. We would like to emphasise again the focus of this paper, which is the comparison between scenarios and the speed of the interactive part of the planning cycle, i.e. the actual device implantation phase. Fourth, mechanical contact analysis should be carried out in the future for more accurate modelling of the vessel wall in response to the deployment of the stent. Fifth, the virtual stenting process can be modelled within a few seconds, but the steady-state CFD computation typically requires 1–1.5 h to complete. A deep learning method such as a physics-informed neural network (PINN) can be integrated into this work to further reduce the CFD computation time. As for more complicated cases such as fusiform aneurysms treated with a peripheral stent using spiral flow technology, transient simulations can be carried out in the future to provide more realistic flow predictions and to facilitate better understanding of potential treatment strategies.

## Conclusions

In this work, a fast virtual stenting algorithm developed in our previous study (Peach et al. 2014a; Spranger and Ventikos 2014; Lyu et al. 2021) was used to deploy different types of endovascular devices into the patient-specific middle cerebral artery (MCA) bifurcation aneurysms. The algorithms proposed previously achieved fast and accurate device deployment which allows for the device positioning and size optimisation. Different deployment strategies and positions may also be tested in future work.

Regardless of the device type, aneurysm inflow is decreased in both of the patient-specific MCA bifurcation aneurysms by at least ~ 85%, and areas of high WSS from flow jetting and impingement are eliminated, all features associated with successful aneurysm isolation. These results suggest that the performance of SVB and Contour device is sufficient for the treatment of these patient-specific bifurcation aneurysms.

The comparison of SVB and Contour device in two cases suggests that the Contour device shows a slightly better inflow reduction ability with an average of 91.25% compared to 87.95% of SVB. However, the placement of SVB shows better side branch WSS magnitude reduction around the placement region. The study on the daughter vessel flow change suggests Contour device deployment increases the daughter vessel flow rate by an average of 11.99%, whereas SVB deployment reduces the daughter vessel flow rate by an average of 8.60%.

## Data Availability

All data generated or analysed during this study are included in the manuscript and supplementary tables and figures. The raw data that support the findings of this study are available from the corresponding author, [YV], upon request.
